# Trigger Thumb in Twins: Case Report

**DOI:** 10.29252/wjps.10.2.110

**Published:** 2021-05

**Authors:** Shohreh Ahmadi, Hossein Akbari, Yousef Shafaei, Peyman Akbari

**Affiliations:** 1 Department of Plastic and Reconstructive Surgery, Iran University of Medical Sciences, Tehran, Iran.; 2 Tehran University of Medical Sciences, Tehran, Iran.

**Keywords:** Trigger thumb, Congenital trigger thumb, Trigger finger

## Abstract

Although trigger finger is common, pediatric trigger thumb is uncommon. In trigger thumb the finger is held in flexed position. The etiology of trigger finger is unknown and can occur isolated without any relation to other syndromes, however there are some evidences that suggest genetic etiology. We reported 2.5 year old twins both having bilateral trigger thumb. Grandfather of the twins had the disease. Although trigger thumb and finger have the same presentation, they can involve different anatomical structures. Bent or straightening of thumb or finger would produce painful popping and clicking and the affected finger or thumb can get stuck in bent and extended position. Based on physical examination and symptoms trigger finger are classified into four stages and each has its own treatment. There are evidences that support congenital hypothesis in pediatric trigger thumb such as bilateral presentation in identical twins, first degree familiar association and etc. Before the 1st year of life, 30% of trigger thumb will get resolved and it is better to postpone the surgery until 2 year of age. A1 pulley release has a good result in pediatric trigger thumb treatment.

## INTRODUCTION

In 1850, Notta introduced Trigger Finger for the first time ^1^. Unlike the adult group, pediatric trigger thumb is an uncommon condition. The etiology of trigger finger and thumb is unknown ^1^^-^^3^. Thumb is held in a fixed flexion position and in examination nodule in Flexor Pollicis Longus (FPL) tendon and Metacarpophalangeal joint (MCPJ) is palpated called Notta node. 

In this article, we present twin girls with trigger thumb of both hands and suggest genetic etiology for that. 

## CASE PRESENTATION

Five-year-old toddler twins with parents attended Hazrat Fateme Clinic, Tehran, Iran of hand surgery in 2019. According to their parents, twins were not able to extend their both thumb freely after playing or catching items and toys, in the last 5 months. 

At first, they could extend both their thumbs with difficulty but later their parents had to help the twins for extension. No other deformity was found. There was not any maternal history of drug use or disease during her pregnancy. Grandfather of twins had the same history of trigger finger. 

Twins had normal growth pattern, and the other fingers were normal. We requested to do soft tissue ultrasonography. Ultrasonography was done and showed hypo echoic region at metacarpophalangeal area, which was site of trigger finger. 

We explained disease treatment plans and possible complications and parents accepted the surgery for their twins. 

**Fig. 1:**
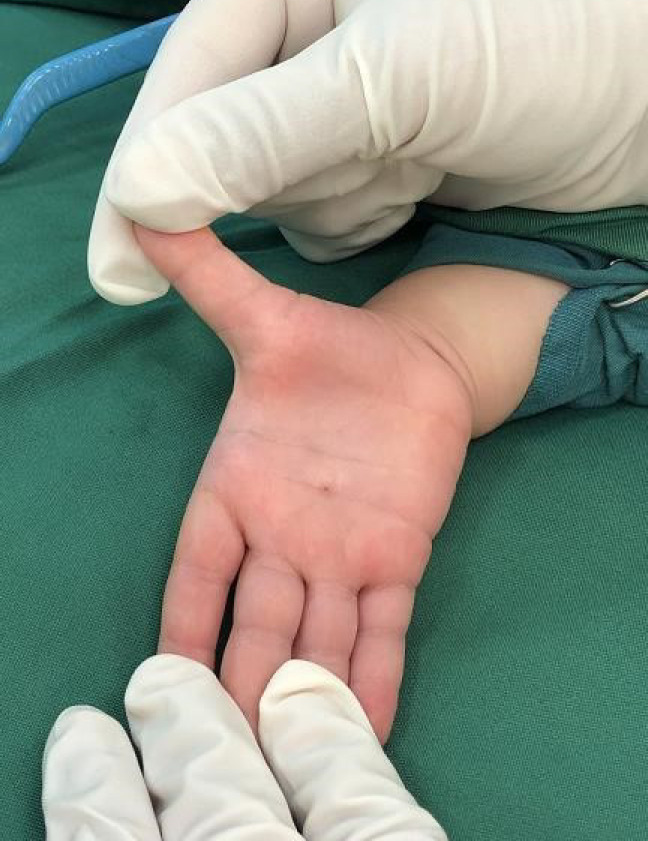
Thumb extension with help

**Fig. 2:**
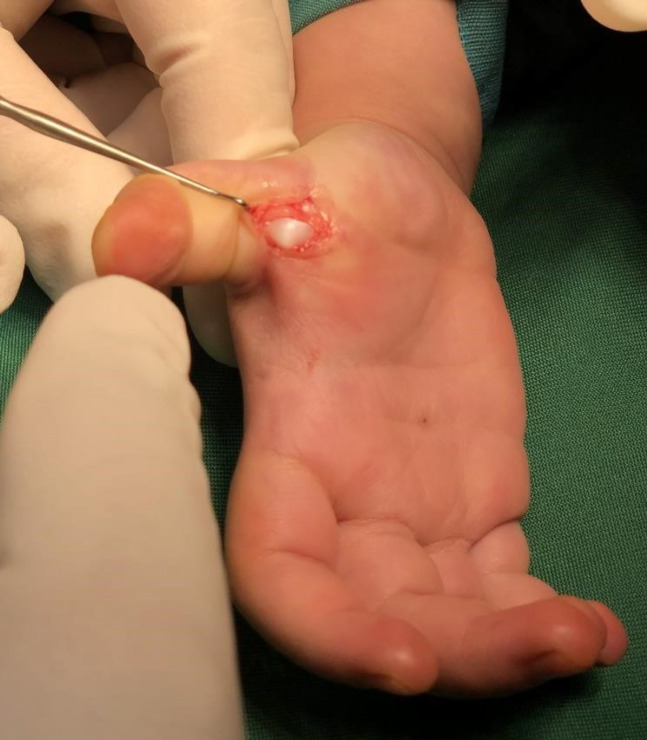
Releasing of the A1 pully

**Fig. 3:**
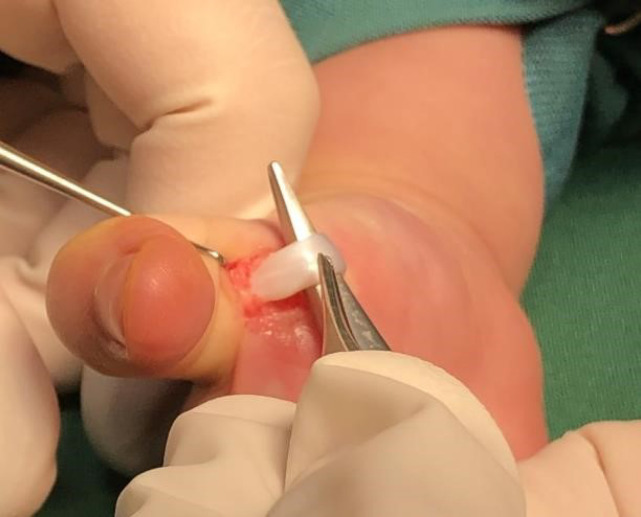
Checking of flexor tendon gliding

**Fig. 4:**
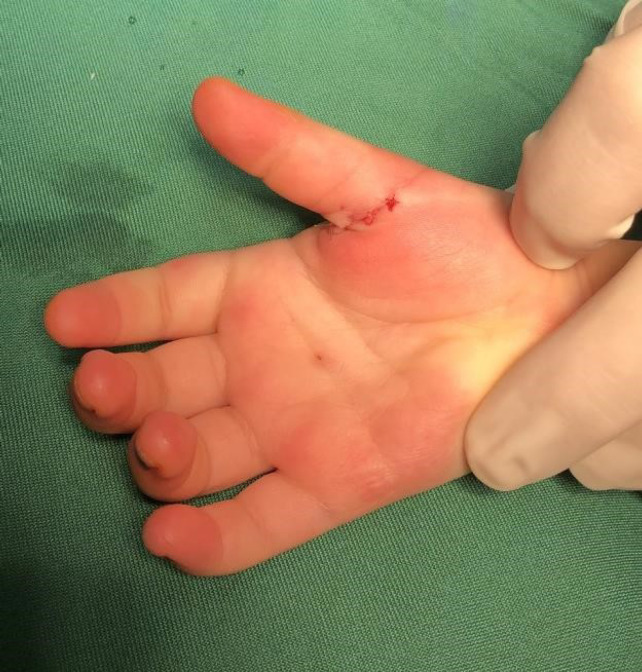
Repairing of skin

After taking written consent and pediatrician and anesthetist consultation, we moved them to the operation room to correct the thumb posture and help the patients to be able to extend their thumbs on their own (Figure 1). Under anesthesia with horizontal incision over MCP Joint on palmar surface A1 pulley release was done and flexor tendon gliding was checked (Figure 2 & 3). After achieving sufficient gliding of tendon, we closed the wound (Figure 4). The same procedure was done for both thumbs and for both toddlers. 

Post operation visit was done in the first week after surgery mother reported that they could flex, and extend their thumbs easily. 

**Table 1:** Stages of trigger finger

**Stage **	**Observation **
1	No triggering in the interphalangeal joint (IPJ) is observed but Notta nodule is present
2	During active extension, triggering is observed
3	During passive extension of IPJ, triggering is observed but active extension of IPJ is not possible
4	There is a fixed flexion deformity and passive extension of IPJ is not possible

## DISCUSSION

Notta first described a series of patients with nodule on tendon in 1850 that interfered with tendons gliding^1^. Trigger thumb is common but not in pediatric group. Incidence of trigger thumb is 3 per 1000 births or less with equal frequency in both males and females^2^. Thumb is affected ten times more than the other fingers^3^. 

Trigger thumb can occur isolated and without relation to any syndromes but neurological syndrome could be accompanied with other finger deformities such as trisomy 18 and Polysacc-haridosis^4^. 

Although the presentation of trigger thumb and finger are similar, they can involve different anatomical structures that require different managements. Trigger thumb is related to A1 pulley constriction but trigger finger other than thumb can be due to A3 pulley, FPL, or FDS thickening^5^. 

The etiology of pediatric trigger thumb is a mismatch between A1 synovial pulley and FPL tendon^6^. 

We can find palpable nodule in FPL at MCPJ level and a fixed flexed position of thumb in examination^2^. 

The palpated nodule in the palmar aspect of trigger finger or thumb is called Notta node and is a bundle of mature collagen and fibroblast^7^. Signs of trigger finger and thumb are included^8^: 

1. The stiffness of affected finger or thumb 

2. Flexing and extending the thumb or finger makes painful popping or clicking 

3. A finger or thumb can get stuck in a flexed or extended position 

4. The symptoms become worse in the morning.

 Clinical observation can help recognize trigger thumb and classify it into four stages^9^^,^^10^. 

Neuromuscular usually preserved^11^. The etiology of trigger finger and thumb is unknown. Inflammation may be the cause of adult trigger finger however; this is not the case in pediatric patient. ^7^ there are still debates, whether trigger finger is acquired or congenital^6^. 

There are no evidence supporting the hypothesis that presentation a trigger thumb in children is down a previous trauma. 

Its differential diagnosis are a congenital loss of extensor tendon, Arthrogryposis, dislocation or fracture of thumb^12^ and injure of thumb ulnar collateral ligament, tendon entrapment in the metacarpal head after trauma^13^^,^^14^. 

When there is a typical presentation of trigger thumb, ultrasonography or MRI may help^13^. A1 pulley release has a good result in pediatric trigger finger^2^. Before one year of age, 30% of trigger thumb will get resolved^8^. Spontaneous resolve of this problem in mild cases is common^2^; for this reason some authors believe the surgery should be postponed until 2 yr of age and they mentioned that there were not any recurrence of nodule or triggering and also any functional deficit^12^. 

In one study, the observation of children between 6 to 30 months of age with trigger finger is done and almost 12% of them had spontaneous resolution^15^. MCPJ hyperextension and loss of IPJ movement can occur in long term^12^. 

Digital never injury, tendon injury, infection, scar contracture and abscess are among the surgical complications however, are not common^11^. 

## CONCLUSION

Trigger thumb is a multifactorial disease without clear pattern but involvement of twins, siblings and generation could be an evidence of genetic effect and hereditary in this problem but further research need to prove this theory. 
